# Conditions in Non-Phthisical Patients Suggesting Tuberculosis of the Lungs

**Published:** 1911-02-11

**Authors:** J. E. Bullock


### The General Practitioner's Column.

[Contributions to this Column are invited, and if accepted, will be paid for.]

### CONDITIONS IN NON-PHTHISICAL PATIENTS SUGGESTING TUBERCULOSIS OF THE LUNGS.

By J. E. BULLOCK, M.D.

Nowadays, in the campaign against consumption, from a desire to detect early cases, there is a likelihood of attributing some minor affection to early consumption. Seeing that such a patient might be treated on sanatorium lines, on insufficient evidence, it is most important that a correct diagnosis should be determined. I propose, in the following remarks, to consider a few symptoms which may suggest a wrong diagnosis—namely, cough and expectoration, shortness of breath, weakness or loss of voice, imperfect development, especially about the chest, bronchitis, hæmoptysis, and general ill-health.

When a patient with such symptoms presents himself, a very careful examination of the chest must be made, so as to detect, if possible, a cause within the chest. If there is expectoration it will, of course, be examined for tubercle bacilli; a negative finding will not exclude lung tuberculosis, and repeated examinations of the sputum must be made. The family history should be weighed carefully. Admitting that phthisis is not directly transmitted, there are certain families in which the disease seems to occur more readily than in others, whether by an inherited diathesis or environment. If *Jeder Mensch ist am Ende ein bischen tuberculose* and 70 to 90 per cent. of all adults have healed scars in their lungs or other evidences of healed tuberculosis, while only 10 per cent. of all deaths are due to the disease, some patients who come before us must have had tubercle in the lungs and yet show no evidence of the disease. Assuming that we find no such evidence, we must take the suggestive symptoms *seriatim*.

### Coughs.

*Cough* may be traceable to irritation of the throat (chronic pharyngitis), connected with post-nasal catarrh, dusty occupations, or abuse of alcohol or tobacco, or dyspepsia. The cough of bronchiectasis may suggest phthisis, especially when it occurs in children. The following conditions serve to distinguish it: it is sudden and paroxysmal and followed by free discharge of purulent fluid, sometimes accompanied by vomiting if there is much straining, or possibly swallowing of the pus; the pus is often not offensive in children. If there is free expectoration from phthisis, signs of cavitation will be present, whereas in bronchiectasis such signs are not distinguishable unless a violent attack of coughing with expectoration has emptied the dilated bronchi. Bronchiectasis in children is very difficult to distinguish from phthisis; in the absence of tubercle bacilli a decision can only be arrived at by considering the history and the physical signs and perhaps by watching the child for a time. Either bronchiectasis or phthisis may follow whooping-cough, broncho-pneumonia, or one of the specific fevers, especially measles. In bronchiectasis the cough will have existed on and off since one of these illnesses. The child is usually better nourished than in phthisis, though if there is no cavitation phthisis may cause but little wasting; the clubbing of the fingers is more marked in bronchiectasis, the lesion is generally one-sided, and though it may involve the whole lung, it is more often at the base; when the result of broncho-pneumonia it is generally double and most marked at the bases; as lobar pneumonia may attack the apex in children, the resulting bronchiectasis at the apex may closely resemble phthisis. In phthisis, with cavitation, there are more signs of secondary infections, such as wasting and elevated temperature. Antecedent pleurisy, not due to chill or accompanying pneumonia, is of great significance, and should always suggest a tubercular origin.

### Adenoids.

Adenoids, with or without enlarged tonsils, are a common cause of cough, consequent on dryness of the throat, through mouth-breathing. As they are often accompanied by contracted chest and bronchitis or collapse of the lung, giving dulness on percussion and feeble breath sounds, the conditions may closely simulate phthisis. In this case a positive diagnosis may be withheld until the adenoids (and tonsils) have been removed. After this the symptoms, if due to adenoids, will disappear. Ozæna and disease of the accessory sinuses of the nose may cause cough, expectoration, and ill-health; the parts must be examined for evidence of such diseases.

*Shortness of breath* is very common in young girls as a result of anæmia; it is not often a marked precursor of phthisis, but may occur with it.

*Weakness or loss of voice* may be due to general muscular weakness or want of tone in the laryngeal muscles from catarrhal inflammation; it may also be due to hysteria, but it must be remembered that adductor or functional paralysis of this nature may occur in a consumptive without any tuberculosis of the larynx. Hoarseness may be due to simple (catarrhal) laryngitis, tubercle, syphilis or carcinoma of the larynx. Simple laryngitis is always bilateral and affects both sides equally, while tubercle, syphilis, or cancer, if not confined to one side, affects one cord more than the other, and may be accompanied by ulceration or a nodular appearance, which is never seen in simple laryngitis.

Points in Diagnosis.

We can here only mention the chief distinctive features which mark out special lesions; in each case the history will largely help the diagnosis. In tuberculous laryngitis the first stage of the affection is an infiltration which affects chiefly the ventricular bands, the posterior parts of the vocal cords, and the inter-arytenoid surface; as it is practically always due to infection by sputum from the lungs, it is a slowly developing disease from pre-existing phthisis: the ulceration which occurs later looks superficial and merges almost imperceptibly into the adjacent healthy mucous membrane. Syphilitic laryngitis is a tertiary lesion, so that there are usually other evidences of the disease; the ulceration is clearly defined, looking punched out and affecting one cord. An early stage of malignant laryngitis shows a swelling and thickening of one cord, which is difficult to distinguish from healthy cord: the early diagnosis of this is the least helped by the history, and the disease is the most remediable by early operation.

In dealing with *hæmoptysis*, the greatest caution must be exercised: in the majority of cases it comes from a deposit in the lungs, which has given no physical signs. If not from the lungs, it may come from the gums, pharynx or the nose: a careful inspection of the parts will detect its source; it is usually scanty, of a brownish colour, not frothy, not intimately mixed with any sputum, and often occurs in the early morning; if from the gums it is associated with carious teeth, if from the pharynx there is local congestion, if from the nose there is a history of nose bleeding. The fact that the patient says it was hawked up without much cough must be accepted with caution, as consumptive patients will often attribute their hæmoptysis to an irritation of the throat.

It is said that 12 per cent. of living children have tuberculosis of the thoracic glands, without implication of the adjacent lung; a diagnosis in this case is difficult unless the glands can be detected by examinations under $x$-rays by a practised observer, or by a repeated low opsonic index. Ailing children with ready tendency to bronchial catarrh and feeble entry of air into the lungs should be placed under good hygienic conditions and carefully watched. Ill health with gastro-intestinal irritation in young persons may suggest phthisis, and may be an early symptom of it; it should be treated by regulating the diet and improving the digestion; in any case, abundance of fresh air will be most helpful.

Conclusions.

In conclusion, I would add a few words on specific tests for tuberculosis. The opsonic index determination requires great skill and accuracy; it is most useful in suspected tuberculosis of thoracic glands accompanied by ill-health: if after a few observations the index is found to be unusually low, such evidence is strongly corroborative of tuberculosis. The ophthalmic reaction is rather too irritating to a sensitive eye, and the result of extended experience among many observers seems to be that it only appears in active lesions, and that some reactions cannot be shown to be tubercular. Von Pirquet's reaction is obtained in many persons over two years of age; it probably shows a pre-existing healed tubercular lesion. A negative reaction repeated is of the most value. We can only arrive at a correct diagnosis of early phthisis, or not, by carefully weighing the significance of the history and environment, by testing the symptoms and physical signs, assisted by the opsonic index and $x$-rays more especially in the case of suspected thoracic glands.

